# A Trimeric HIV-1 Envelope gp120 Immunogen Induces Potent and Broad Anti-V1V2 Loop Antibodies against HIV-1 in Rabbits and Rhesus Macaques

**DOI:** 10.1128/JVI.01796-17

**Published:** 2018-02-12

**Authors:** Andrew T. Jones, Venkateswarlu Chamcha, Sannula Kesavardhana, Xiaoying Shen, David Beaumont, Raksha Das, Linda S. Wyatt, Celia C. LaBranche, Sherry Stanfield-Oakley, Guido Ferrari, David C. Montefiori, Bernard Moss, Georgia D. Tomaras, Raghavan Varadarajan, Rama Rao Amara

**Affiliations:** a Emory Vaccine Center, Yerkes National Primate Research Center, Emory University, Atlanta, Georgia, USA; b Department of Microbiology and Immunology, Emory School of Medicine, Emory University, Atlanta, Georgia, USA; c Molecular Biophysics Unit, Indian Institute of Science, Bangalore, India; d Laboratory of Viral Diseases, National Institute of Allergy and Infectious Diseases, National Institutes of Health, Bethesda, Maryland, USA; e Department of Surgery, Duke University Medical Center, Durham, North Carolina, USA; f Duke Human Vaccine Institute, Duke University Medical Center, Durham, North Carolina, USA; Ulm University Medical Center

**Keywords:** V1V2, V2 hotspot, gp120, human immunodeficiency virus, immunization, rabbits, rhesus macaques, trimeric

## Abstract

Trimeric HIV-1 envelope (Env) immunogens are attractive due to their ability to display quaternary epitopes targeted by broadly neutralizing antibodies (bNAbs) while obscuring unfavorable epitopes. Results from the RV144 trial highlighted the importance of vaccine-induced HIV-1 Env V1V2-directed antibodies, with key regions of the V2 loop as targets for vaccine-mediated protection. We recently reported that a trimeric JRFL-gp120 immunogen, generated by inserting an N-terminal trimerization domain in the V1 loop region of a cyclically permuted gp120 (cycP-gp120), induces neutralizing activity against multiple tier-2 HIV-1 isolates in guinea pigs in a DNA prime/protein boost approach. Here, we tested the immunogenicity of cycP-gp120 in a protein prime/boost approach in rabbits and as a booster immunization to DNA/modified vaccinia Ankara (MVA)-vaccinated rabbits and rhesus macaques. In rabbits, two cycP-gp120 protein immunizations induced 100-fold higher titers of high-avidity gp120-specific IgG than two gp120 immunizations, with four total gp120 immunizations being required to induce comparable titers. cycP-gp120 also induced markedly enhanced neutralizing activity against tier-1A and -1B HIV-1 isolates, substantially higher binding and breadth to gp70-V1V2 scaffolds derived from a multiclade panel of global HIV-1 isolates, and antibodies targeting key regions of the V2-loop region associated with reduced risk of infection in RV144. Similarly, boosting MVA- or DNA/MVA-primed rabbits or rhesus macaques with cycP-gp120 showed a robust expansion of gp70-V1V2-specific IgG, neutralization breadth to tier-1B HIV-1 isolates, and antibody-dependent cellular cytotoxicity activity. These results demonstrate that cycP-gp120 serves as a robust HIV Env immunogen that induces broad anti-V1V2 antibodies and promotes neutralization breadth against HIV-1.

**IMPORTANCE** Recent focus in HIV-1 vaccine development has been the design of trimeric HIV-1 Env immunogens that closely resemble native HIV-1 Env, with a major goal being the induction of bNAbs. While the generation of bNAbs is considered a gold standard in vaccine-induced antibody responses, results from the RV144 trial showed that nonneutralizing antibodies directed toward the V1V2 loop of HIV-1 gp120, specifically the V2 loop region, were associated with decreased risk of infection, demonstrating the need for the development of Env immunogens that induce a broad anti-V1V2 antibody response. In this study, we show that a novel trimeric gp120 protein, cycP-gp120, generates high titers of high-avidity and broadly cross-reactive anti-V1V2 antibodies, a result not found in animals immunized with monomeric gp120. These results reveal the potential of cycP-gp120 as a vaccine candidate to induce antibodies associated with reduced risk of HIV-1 infection in humans.

## INTRODUCTION

While great progress has been made in developing antiretroviral therapies, and preventative measures, such as preexposure prophylaxis, have aided in reducing new infections, an effective vaccine remains crucial to the eradication of human immunodeficiency virus type 1 (HIV-1) (1). Continuous efforts to understand HIV-1 pathogenesis and acquisition have aided in combating the HIV/AIDS pandemic; however, an effective prophylactic vaccine has yet to be developed. As with most conventional vaccines, a strong humoral response is critical for providing protection from infection. Many successful vaccines provide protection by inducing neutralizing antibodies, which act by binding to and inhibiting infectious virions from entering their target cell. However, the generation of broadly neutralizing antibodies (bNAbs) that recognize diverse HIV-1 isolates has been difficult, with nonneutralizing antibodies being the predominant response in both vaccinations and natural infection (2). While uncommon, the spontaneous generation of bNAbs in naturally infected people has been well documented (3, 4), and passive transfer experiments with isolated bNAbs have provided protection in nonhuman primates from simian immunodeficiency virus (SIV)/HIV (SHIV) chimeric infections (5, 6), demonstrating the importance of bNAbs in preventing HIV-1 infection. However, no current HIV-1 vaccine candidate has been able to induce bNAbs in humans.

The main target of antibodies in an HIV-1 vaccine is the HIV-1 Envelope (Env) glycoprotein, which mediates the initial attachment and fusion to host target cells. HIV-1 Env is first expressed as a precursor peptide, gp160, which forms homotrimers before furin-mediated cleavage separates gp160 into its gp120 and gp41 subunits, resulting in a noncovalently linked heterotrimeric spike (7). Monomeric gp120 has been used in multiple HIV-1 vaccine efficacy trials, with many failing to induce broadly cross-reactive neutralizing antibodies and confer protection from infection (8). However, the RV144 vaccine efficacy trial, consisting of an ALVAC-HIV vector prime followed by a monomeric gp120 protein boost, resulted in a short-term vaccine efficacy of 60% and long-term efficacy of 30% (9). This trial demonstrated for the first time the potential for vaccine-induced protection in humans at risk for HIV-1 infection. Surprisingly, protection was not related to the presence or generation of bNAbs. The major correlate of protection was instead found to be nonneutralizing antibodies specific for the scaffolded V1V2 region of gp120 (10, 11), a finding supported by recent nonhuman primate (NHP) trials in which protection from neutralization-resistant SIV infection also correlated with anti-V1V2 antibodies (12–15). Further studies have revealed a key role of V2-targed antibodies in RV144 vaccine-mediated protection (16), as vaccine efficacy was higher against viruses matching the vaccine V2 loop at key residues, and antibodies directed toward linear V2-peptides correlated with lower risk of infection (17, 18). Collectively, these results highlight the need for the development of HIV Env immunogens that induce broadly cross-reactive anti-V1V2 antibodies.

We recently described the design and immunogenicity of a novel trimeric gp120 immunogen in guinea pigs (19). This immunogen, hCMP-v1-cyc-gp120 (referred to as cycP-gp120 here), is based on a cyclically permuted gp120 in which a *de novo* N terminus is generated within the V1 loop region with the native N and C termini joined via an amino acid linker chain (19). To induce a trimeric complex, the human matrix cartilage protein (hCMP) coiled-coil trimerization domain was fused to the *de novo* N termini, resulting in a stabilized, disulfide bond-linked trimeric gp120 (20). Binding studies with monoclonal antibodies showed that cycP-gp120 binds to bNAbs with a higher affinity than gp120, similar to gp140-based trimeric immunogens, including quaternary epitope-specific bNAbs such as PG9, PG16, and PGT145, demonstrating the existence of well-folded trimers (19). Immunization of guinea pigs with cycP-gp120 using a DNA prime/protein boost modality showed induction of neutralizing antibodies against neutralization-resistant (tier-2) HIV-1 isolates. However, the ability of cycP-gp120 immunogen to induce the potentially protective anti-gp70-V1V2 responses is not known. Thus, in this study we tested the immunogenicity of cycP-gp120 protein prime/boost immunizations in rabbits and characterized the neutralizing Ab responses and V1V2 loop-directed responses. In addition, we tested the ability of cycP-gp120 protein to enhance the immunogenicity of our DNA/modified vaccinia Ankara (MVA) vaccines in rabbits and rhesus macaques. Our results show that priming and/or boosting with cycP-gp120 induces high levels of high-avidity HIV-1 Env-specific antibodies, tier-1A and -1B neutralizing antibodies, and the induction of antibody-dependent cellular cytotoxicity (ADCC) activity in MVA-primed rabbits. In addition, we show that cycP-gp120 induces a remarkable anti-gp70-V1V2 antibody response, promoting antibodies recognizing V1V2 sequences from a diverse, multiclade panel of global HIV-1 isolates, as well as antibodies directed toward a core V2-loop region.

## RESULTS

### Trimeric cyclically permuted gp120 induces high levels of high-avidity anti-HIV binding and neutralizing antibodies in rabbits.

**FIG 1:**
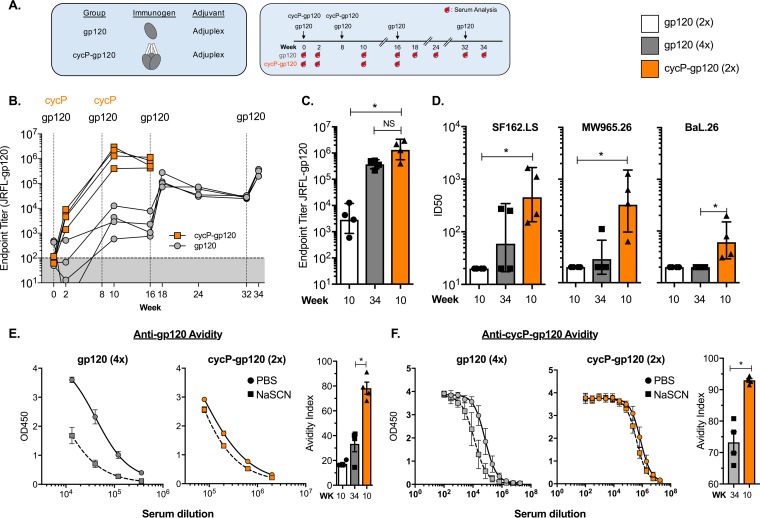
cycP-gp120 induces high titers of high-avidity anti-gp120 antibodies and tier-1A and -1B neutralizing antibodies in rabbits. (A) Schematic of immunogens and trial design. Rabbits were immunized with cycP-gp120 at weeks 0 and 8 or monomeric gp120 at weeks 0, 8, 16, and 32. Serum was collected at weeks 0, 2, 10, and 16 for both groups, and additionally at weeks 18, 24, 32, and 34 for monomeric gp120-immunized rabbits. (B) Kinetics of anti-JRFL gp120-specific IgG titers in serum, measured by endpoint titer. Black dotted lines indicate week of immunization. (C) Comparison of JRFL-gp120-specific IgG titers in serum at 2 weeks after the indicated immunization. (D) Neutralizing antibodies measured using TZM-bl cell-based assays against tier-1A (SF162.LS and MW965.26) and -1B (BaL.26) viral isolates. (E and F) Avidity of JRFL-gp120 (E)- and cycP-gp120 (F)-specific IgG measured using 1.5 M sodium thiocyanate (NaSCN). Avidity index was calculated by measuring the ratio of AUC values of NaSCN to those of PBS-treated samples multiplied by 100. *, *P* < 0.05 by Mann-Whitney test; ID50, dilution of sera required to inhibit 50% of viral infectivity.

To characterize the immunogenicity of cycP-gp120 versus monomeric gp120, we immunized rabbits intramuscularly with 20 μg of either HIV-1 clade B strain JRFL-gp120 or JRFL–cycP-gp120 at weeks 0 and 8 (Fig. 1A). The gp120 group received two additional immunizations at weeks 16 and 32. Sera were collected for both groups at weeks 0, 2, 10, and 16. Additional serum collections for gp120-immunized animals were taken at weeks 18, 24, 32, and 34. Antibodies specific for JRFL-gp120 were determined using enzyme-linked immunosorbent assay (ELISA) in sera taken 2 weeks after the specified immunization. Rabbits receiving two cycP-gp120 immunizations generated a strong anti-JRFL gp120 serum IgG response (geomean titer, 6.1 logs), two logs higher than those of rabbits receiving two monomeric gp120 immunizations (geomean titer, 3.5 logs) (Fig. 1B and C). Impressively, two additional gp120 immunizations were required (geomean titer, 5.6 logs) to generate equivalent JRFL-gp120-specific antibody responses elicited by the two cycP-gp120 immunizations.

Neutralizing antibody responses induced by either gp120 or cycP-gp120 immunization was measured against a multiclade panel of pseudoviruses that have high (SF162.LS and MW965.26), moderate (BaL.26), or low (ADA, JRFL, and TRO.11) neutralization sensitivity (tier-1A, -1B, and -2, respectively). Two cycP-gp120 immunizations generated a strong cross-clade NAb response against tier-1A clade B (SF162.LS) and clade C (MW965.26) viruses (serum dilution required to inhibit viral infectivity by 50% [ID_50_] range, 1.8 to 3.2 logs) (Fig. 1D). In contrast, two gp120 immunizations did not induce detectable NAbs against either tier-1A isolate. The additional two gp120 boosts did generate NAbs against SF162.LS but only in two of the four animals, and only one animal generated neutralizing activity against clade C MW965.26. Immunization with cycP-gp120 also induced NAbs against the more resistant tier-1B clade B BaL.26 virus (ID_50_ range, 1.5 to 2.3 logs; mean ID_50_, 1.6 logs). No neutralizing activity against BaL.26 was observed in gp120-immunized rabbits, and no neutralizing antibodies against tier 2 pseudoviruses, including JRFL, were observed in any of the immunized animals (Table 1). However, it is possible that additional immunizations with cycP-gp120 help to generate these responses, as previous studies found that repeated germinal center reactions aid in the generation of autologous tier-2 neutralization activity (21–23).

**TABLE 1:**
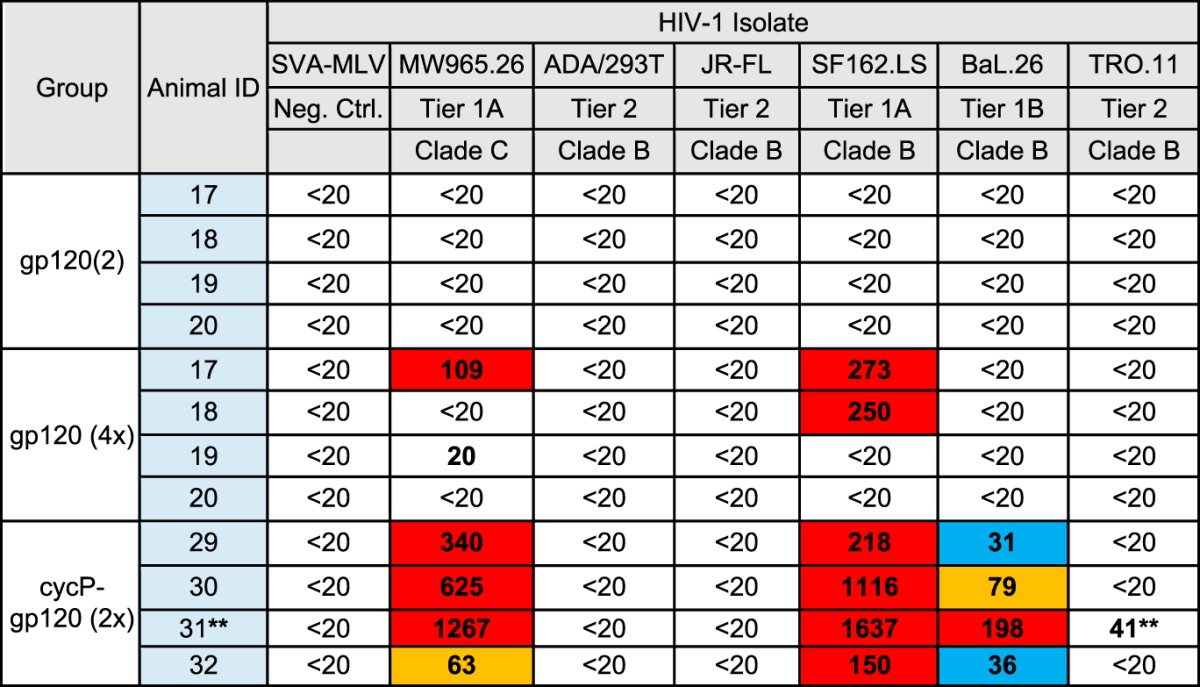
Neutralizing antibody titers againsttier-1A, -1B, and -2 HIV-1 isolates^*a*^

a Neutralizing antibodies generated by gp120 (2×), gp120 (4×), or cycP-gp120 (2×) immunization in rabbits measured by TZM-bl assay against a panel of tier-1A, -1B, and -2 HIV-1 isolates. Data are reported as ID_50_, i.e., dilution of sera required to inhibit 50% of viral infectivity. Positive values are reported as >3× the background signal against SVA-MLV. **, Cell toxicity noted in first two dilutions of animal 31 with all viruses. Titers of up to 1:180 may not reflect true neutralizing activity.

Antibody avidity plays an important role in protection from HIV/SIV infection (24–28). To measure antibody avidity against JRFL-gp120, we performed an ELISA in which rabbit sera bound to JRFL-gp120 was incubated with 1.5 M sodium thiocyanate (NaSCN), which dissociates weakly bound antibodies, or phosphate-buffered saline (PBS) prior to detection. Rabbits immunized twice with gp120 had low-avidity anti-JRFL gp120 antibodies (mean avidity index of 17.3), which modestly increased upon subsequent gp120 immunizations (mean avidity index of 33.4) (Fig. 1E). However, immunization with cycP-gp120 resulted in very-high-avidity antibodies (mean avidity index of 78.2) that were markedly higher than those of animals immunized with monomeric gp120. As cycP-gp120 is antigenically distinct from monomeric gp120 as well as a trimeric protein, we also tested antibody avidity against cycP-gp120. As with monomeric gp120, rabbits immunized with cycP-gp120 generated very-high-avidity antibodies against cycP-gp120 (mean avidity index of 93), significantly higher than that of gp120-immunized rabbits (mean avidity index of 73) (Fig. 1F). Taken together, these data suggest that cycP-gp120 is a robust HIV-1 Env immunogen capable of generating very high titers of high-avidity antibodies, as well as tier-1A and -1B neutralizing antibodies, after only two immunizations in rabbits.

### cycP-gp120 induces a robust antibody response against V1V2 variable loops derived from global HIV-1 isolates.

Recent vaccine studies in both nonhuman primates and humans have emphasized the importance of antibodies targeting the V1V2 loop region of gp120 in protecting from HIV-1 infection (10, 12–14). To test for the presence of binding antibodies to diverse HIV-1 isolates, we measured binding to gp120, gp140, and gp70-V1V2 scaffold proteins derived from a global panel of tier-2 HIV-1 isolates from acute/early infection representing multiple clades of HIV. The gp70-V1V2 scaffold proteins included case A2 and C.1086 strains, as the presence of binding Abs to these proteins was shown to be associated with reduced risk of infection in the RV144 trial (11).

**FIG 2:**
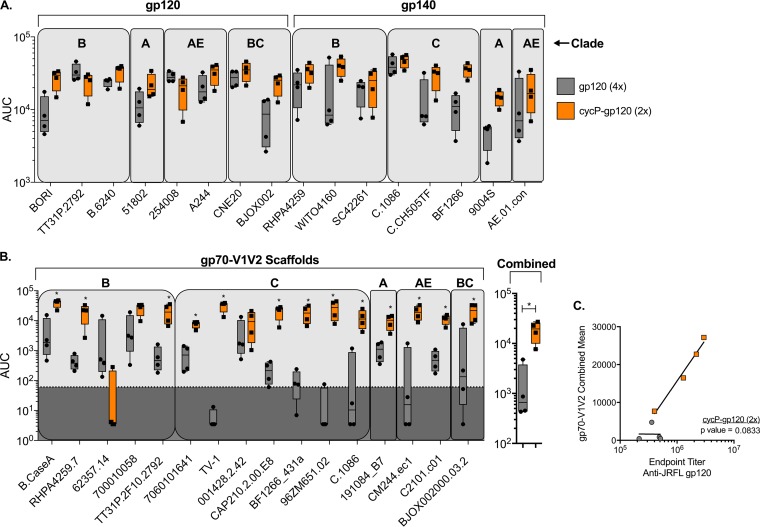
Binding antibody multiplex assay (BAMA) analysis of rabbit immune serum reactivity to a global panel of HIV-1 gp120, gp140, and gp70-V1V2 scaffolds. Serial dilutions of sera from rabbits immunized with gp120 (4×) or cycP-gp120 (2×) were reacted against a multiclade panel of gp120/gp140 (A) and gp70-V1V2 (B) scaffold proteins and detected by a BAMA. Data are reported as AUC analysis values. Individual animal data points are plotted over box-and-whiskers plots. Combined AUC values were measured by calculating the average AUC for each animal against all gp70-V1V2 scaffold proteins. Shaded area represents threshold for positive reactivity. (C) Correlation analysis of gp70-V1V2 combined AUC of each immunized animal with their respective anti-JRFL endpoint titers (Fig. 1C). *, *P* < 0.05 by Mann-Whitney test and Spearman correlation test.

Rabbits immunized twice with cycP-gp120 or four times with gp120 showed comparable binding to gp120/gp140 proteins from multiple isolates, including cross-clade reactivity against non-clade B isolates (Fig. 2A). The cycP-gp120-immunized animals showed higher binding activity against some isolates and lower binding activity against others compared to animals immunized with four gp120 immunizations. These results demonstrate that both monomeric gp120 and trimeric cycP-gp120 induce comparable titers of binding antibody response against gp120/gp140 proteins from a diverse panel of HIV-1 isolates. However, in sharp contrast to binding to gp120/gp140 proteins, rabbits immunized twice with gp120 showed no detectable responses against gp70-V1V2 scaffolds (data not shown), and further gp120 immunizations resulted in only a weak response, with 9 out of 16 tested isolates being recognized by all vaccinated animals, suggesting that monomeric JRFL gp120 elicits poor V1V2 scaffold-directed antibody responses even after multiple immunizations. Impressively, two cycP-gp120 immunizations resulted in a robust induction of anti-V1V2 antibodies against 15 of the 16 isolates tested (Fig. 2B). A strong trend was observed between the overall anti-gp120 response and V1V2-directed antibodies in cycP-gp120-immunized rabbits, a correlation not found in gp120-immunized rabbits (Fig. 2C), indicating that cycP-gp120 can preferentially promote V1V2 antibodies as part of the overall antibody response against HIV-1 Env. These results demonstrate that cycP-gp120 serves as an excellent immunogen to induce broadly cross-reactive antibody response against V1V2 scaffolds from diverse HIV-1 isolates from multiple clades.

### cycP-gp120-induced antibody response is targeted against the V2 hot-spot peptide.

**FIG 3:**
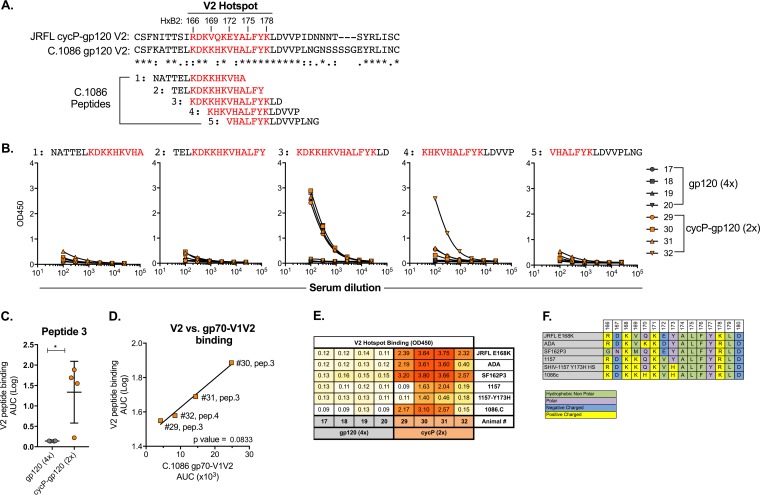
V2-directed antibodies generated by cycP-gp120. (A) Alignment of V2 regions of cycP-gp120 and C.1086 gp120. V2 hot spot is indicated in red. (B) Overlapping 15-mer peptides derived from C.1086 were reacted against sera from rabbits immunized with gp120 (4×) or cycP (2×), and antibody binding was detected by ELISA. Lines represent individual animals, and animal numbers are indicated. (C) AUC analysis of binding to peptide 3. (D) Correlation analysis of gp70-V1V2 1086C scaffold binding (Fig. 2B) for each cycP-gp120-immunized animal with their respective binding to the indicated V2 peptide. Rabbits 29, 30, and 31 are matched against peptide 3. Rabbit 32 is matched against peptide 4. (E) V2 hot-spot binding to peptides derived from different HIV-1 strains, detected by ELISA binding and measured by OD_450_. (F) Sequences of V2 hot-spot peptides used for panel E. *, *P* < 0.05 by Mann-Whitney test and Spearman correlation test.

The generation of cross-clade binding antibodies to gp70-V1V2 scaffolds suggests that these antibodies are targeted against a conserved epitope in the V1V2 region or against a specific conformation found on these scaffolds. Recent studies have identified V2-directed antibody responses as a correlate of protection in the RV144 trial, with antibodies targeting a core hot-spot region within the V2 loop associating with a decreased risk of infection (17). This region corresponds to residues 166 to 178 (strain HxB2) of gp120, a region proximal to the putative α_4_β_7_ binding site motif (29). To test for the generation of cross-clade-reactive V2-targeting antibodies, we measured binding of rabbit sera to five 15-mer peptides overlapping by 12 amino acids corresponding to the V2 hot-spot region of the clade C.1086 HIV-1 gp120 (Fig. 3A). The hot-spot region of C.1086 shares 61% identity to clade-B JRFL sequence. Surprisingly, despite incomplete homology, sera from rabbits immunized with cycP-gp120 showed strong binding to two of the five peptides tested, with three of the four animals binding to a core peptide encoding the entirety of the hot-spot region and one rabbit responding to the subsequent overlapping peptide (Fig. 3B). In contrast, no measurable binding activity was detected in rabbits immunized with monomeric gp120 (Fig. 3C). Furthermore, we found a direct association (trend) between binding to V2 peptides (peptide 3 or 4) and overall binding to the C.1086 gp70-V1V2 scaffold (Fig. 3D).

To further characterize the V2 hot-spot-directed response, we synthesized V2 hot-spot peptides from multiple clade B and clade C viral strains that represent the immunogen strains (JRFL and ADA) and potential SHIV strains (clade-B SF162P3, clade-C 1157, and 1157-Y173H, which are currently being used in nonhuman primate vaccine protection studies) (30). As with C.1086, cycP-gp120-immunized rabbits developed a broad anti-V2 hot-spot antibody response to multiple V2 hot-spot peptides (Fig. 3E). Very low or no reactivity was found in gp120-immunized rabbits against any of the peptides. The V2 peptide sequences show great diversity and differ from the JRFL vaccine strain at several residues (Fig. 3F), suggesting that V2 hot-spot-directed antibodies induced by cycP-gp120 are flexible and capable of binding to multiple diverse HIV-1 isolates. As V2-directed antibody responses have been shown to correlate with protection in both human and nonhuman primate vaccine studies, the ability of cycP-gp120 to induce a strong cross-reactive V2 hot-spot response greatly adds to its potential as a vaccine candidate.

### cycP-gp120 serves as a strong booster immunization in rabbits primed with MVA-HIV.

The results from the VAX003 and RV144 trials suggest that the implementation of a poxvirus vector prime in the RV144 trial is important for promoting protective immune responses, and recent studies suggest these responses can be attributed to the promotion of distinct IgG subclasses between the two trials (14, 31). There is now a concerted effort in the HIV-1 vaccine field to evaluate the use of protein booster immunizations in viral vector priming immunizations, in particular for protein immunogens that strongly induce gp70-V1V2 responses. In light of this, we evaluated the ability of cycP-gp120 to boost humoral immune responses primed by another recombinant poxvirus, modified vaccinia Ankara (MVA), engineered to express trimeric membrane-bound gp150 derived from HIV-1 clade B strain ADA (MVA-HIV) (32) on infected cells and virus-like particles, a vector currently being evaluated in human trials (33).

**FIG 4:**
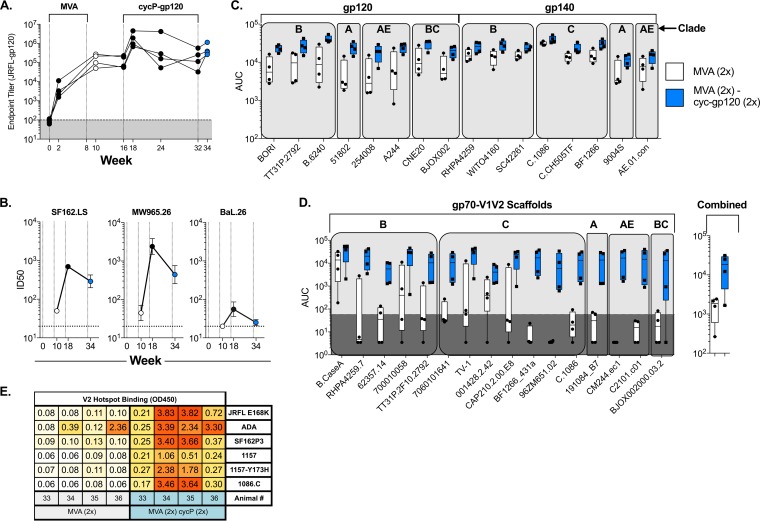
Boosting MVA-primed rabbits with cycP-gp120 promotes neutralizing antibodies, increased gp120/gp140, and gp70-V1V2-directed antibodies. Rabbits were immunized twice with MVA-HIV at weeks 0 and 8 and boosted with cycP-gp120 protein at weeks 16 and 32. (A) Kinetic analysis of JRFL-gp120-specific IgG endpoint titers measured via ELISA. (B) Neutralizing antibodies measured at weeks 10, 18, and 34 against tier-1A and -1B viruses, reported as ID_50_ concentrations. (C and D) BAMA analysis of sera from weeks 10 and 34 against gp120/gp140 proteins (C) and gp70-V1V2 scaffold proteins (D), reported as AUC analysis values. Shaded area represents threshold for positive reactivity. (E) V2 hot-spot binding, detected by ELISA, from week 10 and week 34 sera.

To test the use of MVA-HIV and cycP-gp120 in a poxvirus prime/protein boost scheme, we immunized rabbits intramuscularly with MVA-HIV at weeks 0 and 8 (32), followed by cycP-gp120 immunizations at weeks 16 and 32. Two MVA-HIV immunizations induced strong binding antibodies against JRFL-gp120 (mean titer, 5.1 logs), and these responses were boosted by approximately 1 log upon subsequent immunization with cycP-gp120 before contracting over the next 14 weeks before a final cycP-gp120 boost (mean titer, 5.7 logs) (Fig. 4A). Neutralizing antibody responses were measured at week 10, after the second MVA-HIV immunization, week 18, after the first cycP-gp120 boost, and week 34, after the final cycP-gp120 boost. Encouragingly, boosting once with cycP-gp120 expanded both tier-1A and -1B neutralizing antibodies (Fig. 4B). However, the second protein boost did not efficiently recall the antibody response, and thus the neutralizing antibody titers also were not boosted following the second protein boost (Fig. 4B). Analysis at week 34, 2 weeks after the second cycP-gp120 immunization, showed an expansion of binding to the global panel of HIV-1 gp120/gp140 proteins, highlighting the ability of cycP-gp120 to promote and expand antibodies with multiclade breadth (Fig. 4C). While MVA-HIV immunization induced strong gp120 binding antibodies, responses against gp70-V1V2 scaffolds were much weaker, with only 1 out of 16 isolates tested, Case.A2, showing 100% positive binding in MVA-HIV-immunized animals. However, boosting with two cycP-gp120s greatly expanded both the magnitude and breadth of gp70-V1V2-directed responses, with 15 out of 16 isolates showing positive binding by all animals (Fig. 4D), demonstrating cycP-gp120's propensity for inducing V1V2-directed antibody responses. As with rabbits immunized only with cycP-gp120, boosting MVA-primed rabbits with cycP-gp120 also promoted the generation of V2 hot-spot binding antibodies, responses not observed after the initial MVA-HIV immunizations (Fig. 4E). These data show that cycP-gp120 acts as a strong boosting immunogen in MVA-HIV-primed animals, promoting both V1V2-scaffold- and V2 hot-spot-directed responses.

**FIG 5:**
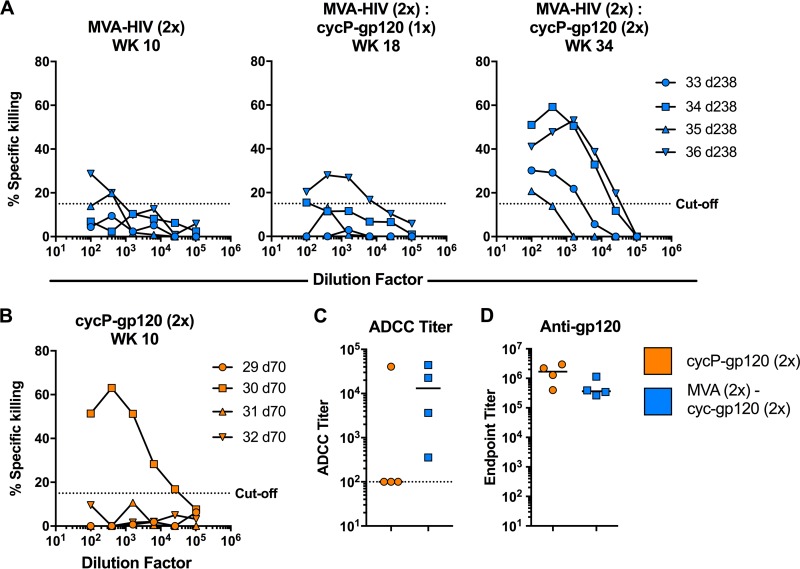
ADCC activity in rabbits immunized with cycP-gp120 or MVA-HIV prime/cycP-gp120 boost. ADCC activity against JRFL-infected target cells measured against sera from weeks 10, 18, and 34 in rabbits immunized with MVA-HIV followed by boosting with cycP-gp120 (A) and week 10 from rabbits immunized with cycP-gp120 alone (B). Percent specific killing values were subtracted from week 0 sera to remove background ADCC activity. ADCC titer (C) and anti-gp120 serum IgG titer (D) comparison between cycP-gp120 (2×)- and MVA-HIV (2×)- and cycP-gp120 (2×)-immunized rabbits. ADCC Ab titers were defined as the reciprocal of the highest dilution indicating a positive response.

A major effector function of nonneutralizing antibody responses is antibody-dependent cellular cytotoxicity (ADCC), and recent studies examining antibody responses from the RV144 trial suggest that ADCC is involved with vaccine efficacy (14). To measure ADCC activity in rabbits, we cocultured HIV-1 JRFL-infected target cells with effector NK cells and serial dilutions of rabbit sera taken at different time points. While MVA-HIV immunization led to a low level of ADCC activity, this activity was boosted after the two cycP-gp120 boosts (Fig. 5A), with all four rabbits developing ADCC activity against HIV-1-infected cells. In contrast, immunization with cycP-gp120 alone resulted in only one animal out of four developing ADCC activity (Fig. 5B), despite having similar if not higher levels of anti-JRFL gp120 serum IgG titers than MVA-HIV-primed, cycP-gp120-boosted rabbits (Fig. 5C and D). Additionally, both groups have strong V1V2-directed breadth and JRFL-gp120 V2 hot-spot-directed antibodies. While the difference in ADCC titers in animals immunized with cycP-gp120 or MVA-HIV prime/cycP-gp120 boost does not reach statistical significance, these data suggest that the development of ADCC activity is not based solely on the magnitude or specificity of the antibody response. Additionally, these data suggest that the utilization of a poxvirus vector prime prior to a protein boost could aid in the development of ADCC functionality.

### cycP-gp120 serves as a strong booster immunization to MVA-primed antibody responses in rhesus macaques.

**FIG 6:**
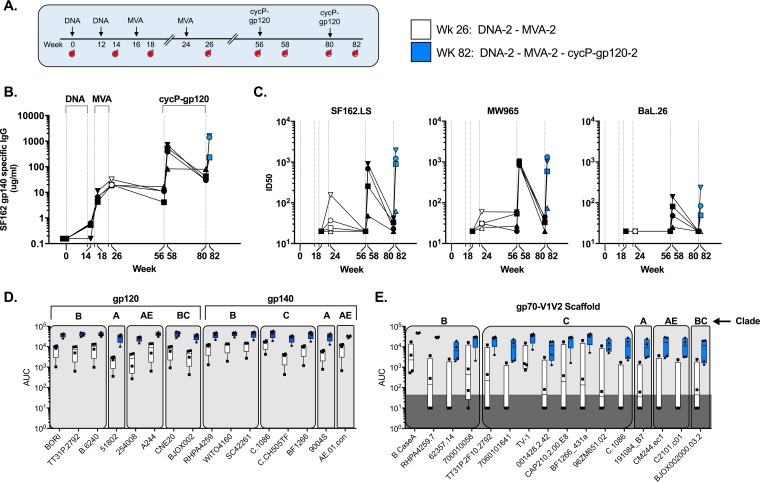
Boosting rhesus macaques primed with a DNA/MVA vaccine with cycP-gp120. (A) Study design. Four rhesus macaques were immunized with DNA expressing HIV-1 gag/pol/env at weeks 0 and 12 and boosted with MVA-HIV at weeks 16 and 24 before boosting with recombinant JRCSF cycP-gp120 protein at weeks 56 and 80. (B) Kinetic analysis of serum IgG specific for HIV-1 SF162 gp140 (μg/ml). (C) Neutralizing antibodies against tier-1A (SF162.LS and MW965) and -1B (BaL.26). (D) BAMA analysis of week 26 and week 82 sera against a global panel of HIV-1 gp120, gp140, or gp70-V1V2 (E) scaffold proteins, reported as AUC values.

Our clade B DNA/MVA vaccine has completed phase 2a immunogenicity studies in humans, and plans are under way for phase 2b efficacy studies (34). To test the immunogenicity of cycP-gp120 in the nonhuman primate model, we vaccinated four rhesus macaques intramuscularly with our clade B DNA/MVA HIV vaccine, followed by two subcutaneous boosts with HIV-1 JRCSF cycP-gp120 (Fig. 6A). Similar to our results in rabbits, cycP-gp120 served as a potent immunogen in rhesus macaques, boosting both gp140-specific serum IgG as well as neutralizing antibodies against tier-1A and -1B HIV-1 isolates, with neutralizing antibodies against the moderately resistant HIV-1 BaL.26 being detected only after boosting with cycP-gp120 (Fig. 6B and C). Boosting with cycP-gp120 enhanced the magnitude of cross-reactive antibodies specific for gp120 and gp140 antigens and greatly expanded the global gp70-V1V2 response, with all animals reacting against all scaffolds tested after the second protein boost (Fig. 5D and E). Taken together, these data show that cycP-gp120 can serve as a potent vaccine immunogen in rhesus macaques, warranting future studies to further examine its efficacy in HIV/SHIV challenge studies.

## DISCUSSION

Recent efforts in HIV-1 vaccine design have focused on the design and implementation of novel trimeric HIV-1 Env immunogens with the goal of generating protective antibody responses, such as bNAbs (35) and V1V2-directed antibodies (36). In this study, we characterized the immunogenicity of a trimeric cycP-gp120 protein in rabbits and rhesus macaques and demonstrate that cycP-gp120 is a robust HIV-1 Env immunogen capable of generating high titers of high-avidity HIV-1 Env-specific antibodies, tier-1A and -1B neutralizing antibodies, and broadly cross-reactive V1V2 and V2 hot-spot-directed antibodies, and it induces ADCC activity in MVA-primed rabbits. These results highlight the potential of cycP-gp120 protein to serve as an optimal protein immunogen for homologous and heterologous prime/boost HIV-1 vaccines.

A striking feature of cycP-gp120 is the ability to induce broadly cross-reactive V1V2-directed antibody responses, a major correlate of decreased HIV-1 risk in the RV144 trial (10, 11, 31), measured against a global panel of multiclade HIV-1 gp70-V1V2 scaffolds. Furthermore, we show that cycP-gp120 induces heterologous, cross-clade-reactive antibodies targeting specific regions of the V2 loop that are associated with decreased risk of infection (16). The mechanisms by which anti-V1V2 antibodies mediate protection from HIV-1 infection are not completely understood; however, it was hypothesized that nonneutralizing effector functions such as ADCC and Fc-mediated phagocytosis are important. The lack of ADCC activity in the cycP-gp120-immunized rabbits despite high V1V2 levels suggests that V1V2-directed antibody responses alone are not an indicator of ADCC potential. cycP-gp120 (alone)-immunized and MVA-HIV-primed/cycP-gp120-boosted rabbits have similar levels of V1V2-directed breadth and JRFL-V2 hot-spot-directed antibody responses, yet MVA-HIV-primed/cycP-gp120-boosted rabbits generated enhanced ADCC activity against HIV-1 JRFL-infected target cells, suggesting some other parameters are necessary for ADCC activity.

While ADCC is thought to be a potential function in which nonneutralizing antibodies aid in protection from infection, there have been other proposed mechanisms by which these antibodies act. It has been reported that the V2 loop of HIV-1 gp120 binds to the integrin α_4_β_7_ expressed on activated CD4^+^ T cells, mediating viral uptake and infection (29, 37), which could be abrogated by anti-V1V2 antibodies, although this may not be applicable for all HIV-1 strains (38). As discussed above, multiple studies have demonstrated the importance of the V2 region for generating protective antibody responses. Alignment analysis of the gp70-V1V2 scaffolds tested in this study show that the V2 region proximal to the beta-strand C of the V1V2 region is generally more conserved than the hypervariable V1 loop, suggesting that the cross-reactive gp70-V1V2 binding antibodies induced by cycP-gp120 are focused to this more conserved region in V2 (data not shown). Furthermore, the design of cycP-gp120 involves the introduction of hCMP trimerization domain within the V1 loop, which may result in focusing the antibody response away from the V1 loop to the V2 region (19).

One of the potential issues with gp41-containing HIV-1 Env immunogens in humans is the induction of gp41-biased antibody responses. Thus, a major limitation of gp140-based immunogens, including the SOSIP versions, could be the strong induction of gp41-specific binding antibody responses, as they may promote cross-reactive antibodies specific for host proteins as well as microbiota-derived antigens (39). A key feature of cycP-gp120 is the lack of the gp41 domain, and we predict that immunization with cycP-gp120 in humans would not induce and potentially diverge the antibody response toward these nonprotective epitopes and instead would promote antibody responses to relevant epitopes found on the outer domain of gp120. Additionally, antibody responses primed by viral vectors presenting full-length gp160 would preferentially be boosted toward gp120-localized epitopes rather than gp41 epitopes in a prime/boost vaccination model, which could result in an overall more protective antibody response.

Currently, most soluble trimeric HIV-1 Env protein immunogens are based on HIV-1 Env gp140, a modified version of gp160 lacking the transmembrane domain of gp41. Most notably, the SOSIP.664 gp140 trimer has been well characterized as a properly formed trimeric gp140 immunogen that displays epitopes for bNAbs, and recent work has shown induction of autologous tier-2 neutralizing antibodies in nonhuman primates (23, 40, 41). While immunization with cycP-gp120 trimer induced tier-1B neutralizing antibodies, we did not see generation of tier-2 neutralizing antibodies. However, our previous studies with cycP-gp120 in guinea pigs did show induction of tier-2 neutralizing antibodies (19). This discrepancy could be due to differences in immunization regimen, lack of adequate number of immunizations, or the species used. In the guinea pig study, we used two DNA primes and two protein boosts with cycP-gp120, whereas in the current study we used only two cycP-gp120 protein boosts. Indeed, recent evidence indicates that repeated germinal center reactions during multiple immunizations help with the generation of neutralizing antibodies (21). In our study, neutralizing activity was often developed in conjunction with a strong boost in the overall anti-gp120 binding antibody response, suggesting that the generation of tier-1A and -1B neutralizing antibodies is a consequence of the overall increase in the magnitude of the response. However, rabbits immunized with monomeric gp120 did not develop tier-1B neutralizing antibody responses, even after repeated immunizations and titers similar to those of cycP-gp120-immunized rabbits, indicating that the anti-Env titers alone do not predict neutralizing activity.

It is important to note that cycP-gp120-based trimeric immunogens provide multiple advantages over SOSIP or NFL gp140 trimeric immunogens. First, since nearly 100% of the expressed protein exists in the trimeric form, purification can be done in a single step using lectin affinity compared to multiple laborious steps required to purify gp140 trimers. As a result, the protein yields will be significantly greater with cycP-gp120 immunogens, which is a critical factor for clinical translation. Second, as described above, cycP-gp120 immunogens lack the gp41 region and thus can avoid gp41-dominant antibody response in humans. Third, we do not yet know if gp140 trimeric immunogens can induce a broadly cross-reactive anti-V1V2 antibody response or antibody response against the V2 hot-spot region or generate ADCC activity against HIV-infected cells.

The vaccine-mediated protection seen in the RV144 trial demonstrated the effectiveness of the poxvirus prime/protein boost vaccine strategy over a protein-alone vaccine used in the Vaxgen trial (14). In this study, we show that cycP-gp120 is an effective boosting immunogen in rabbits with poxvirus (MVA-HIV) vectors, promoting neutralizing antibodies, broad V1V2 breadth, V2 hot-spot-directed antibodies, and ADCC activity. Similarly, in rhesus macaques, cycP-gp120 acts as a potent boosting immunogen, inducing neutralizing antibodies and desirable V1V2-directed antibody responses. Going forward, it will be important to compare cycP-gp120 to other promising trimeric protein immunogens, such as SOSIP and NFL versions that can induce autologous tier-2 neutralizing antibody responses. In addition, it would also be important to test the effects of other recently described Env trimer-stabilizing mutations in the cycP-gp120 background on its immunogenicity. Nevertheless, given our highly encouraging immunogenicity results combined with its relative ease of purification, we believe cycP-gp120 has great potential as a vaccine candidate. Thus, we believe these results warrant further studies to characterize cycP-gp120 in nonhuman primate challenge models.

## MATERIALS AND METHODS

### Ethics statement.

All housing and experiments involving rhesus macaques were conducted at the Yerkes National Primate Research Center, and protocols were approved by the Emory University Institutional Animal Care and Use Committee (IACUC), protocol YER-2002080. Experiments were carried out in accordance with USDA regulations and recommendations derived from the *Guide for the Care and Use of Laboratory Animals* (42). Rhesus macaques were housed in pairs in standard nonhuman primate cages and provided with standard primate feed (Purina monkey chow), fresh fruit, and enrichment daily, as well free access to water. Immunizations, blood draws, and other sample collections were performed under anesthesia with ketamine (5 to 10 mg/kg of body weight) or telazol (3 to 5 mg/kg) and were performed by trained research staff. Rabbits were housed and immunized by Bioqual Inc. (Rockville, MD) in accordance with IACUC protocol 14-R133.

### Animals and immunizations. (i) Rabbits.

Twelve female (4 animals/group) New Zealand White rabbits (age, 10 to 12 weeks) were immunized intramuscularly with 20 μg of recombinant JRFL-gp120 (weeks 0, 8, 16, and 32) or JRFL-hCMP-V1cyc-gp120 (cycP-gp120) (weeks 0 and 8). Both JRFL-gp120 and cycP-gp120 contain the E168K mutation. An additional group was immunized intramuscularly with 1 × 10^8^ PFU MVA-62BSm (weeks 0 and 8), followed by boosting with cycP-gp120 (weeks 16 and 32). JRFL-gp120 and cycP-gp120 immunizations were given with the adjuvant Adjuplex (Sigma). The production of cycP-gp120 was as described previously (19). JRFL-gp120 was a kind gift from Richard Wyatt at the Scripps Research Institute.

### (ii) Rhesus macaques.

Four Indian rhesus macaques were immunized intramuscularly with two DNA primes (weeks 0 and 12) followed by two MVA-62Bsm boosts (weeks 16 and 24), with both vaccines encoding HIV-1 clade B HxB2 gag/pol and ADA env. In addition, animals were boosted subcutaneously with 100 μg of JRCSF-cycP-gp120 with Adjuplex at weeks 56 and 58.

### ELISA and avidity assays.

Rabbit serum IgG binding to recombinant JRFL-gp120 was measured using JRFL-gp120 (Immune-Tech)-coated ELISA plates and reacted against serial dilutions of sera before detection with anti-rabbit IgG horseradish peroxidase (HRP) (Southern Biotech). Endpoint titers were measured by calculating the serum dilution required to reach an optical density (OD) of 0.16, four times the background level of detection. Antibody avidity was calculated by incubating JRFL-gp120- or cycP-gp120-coated ELISA plates with serial dilutions of rabbit sera before incubation for 10 min with PBS or 1.5 M sodium thiocyanate (NaSCN). Plates then were washed and bound antibodies detected using anti-rabbit IgG HRP. The avidity index was determined by measuring the ratio of the area under the concentration-time curve (AUC) for PBS- or NaSCN-treated sera. For rabbit sera binding to V2 peptides, 15-mer peptides corresponding to the HIV-1 C.1086 V2 region were synthesized (Genemed Synthesis Inc.) and diluted in PBS before coating onto ELISA plates (1 μg/ml). Serial dilutions of rabbit sera were reacted against V2 peptides, and AUC analysis was used to measure binding. Additional peptides corresponding to the V2 hot-spot region of HIV-1 strains JRFL-E168K (N-RDKVQKEYALFYKLD-C), ADA (N-RDKVKKDYALFYRLD-C), SF162P3 (N-GNKMQKEYALFYRLD-C), 1157 (N-RDKKQKVYALFYRLD-C), and 1157-Y173H (N-RDKKQKVHALFYRLD-C) (30) were synthesized (Genemed Synthesis Inc.), and binding by rabbit sera diluted 1:100 was measured by OD_450_ reading. Rhesus macaque serum IgG binding to recombinant HIV-1 SF162 (clade B) gp140 (Immune-Tech) was measured by ELISA against serial dilutions of sera and detected with anti-rhesus IgG HRP (Southern Biotech) before quantification against a standard curve against rhesus IgG.

### Neutralization assays.

Neutralizing antibody activity was measured in 96-well culture plates by using Tat-regulated luciferase (Luc) reporter gene expression to quantify reductions in virus infection in TZM-bl cells. TZM-bl cells were obtained from the NIH AIDS Research and Reference Reagent Program, as contributed by John Kappes and Xiaoyun Wu. Assays were performed with HIV-1 Env-pseudotyped viruses as described previously (43). Test samples were diluted over a range of 1:20 to 1:43,740 in cell culture medium and preincubated with virus (∼150,000 relative light unit [RLU] equivalents) for 1 h at 37°C before addition of cells. Following 48 h of incubation, cells were lysed and Luc activity determined using a microtiter plate luminometer and BriteLite plus reagent (PerkinElmer). Neutralization titers are the sample dilution at which RLU were reduced by 50% compared to RLU in virus control wells after subtraction of background RLU in cell control wells. Serum samples were heat inactivated at 56°C for 1 h prior to assay. Positive values were reported as being at least 3× baseline values standardized against the negative-control virus, SVA-MLV.

### BAMA measuring gp120, gp140, and gp70-V1V2 scaffold binding.

Binding antibody multiplex assays (BAMA) were performed as described previously (10, 44). Briefly, serial dilutions (starting at 1:80, six 5-fold dilutions) of rabbit or rhesus macaque sera were reacted against beads conjugated to a panel of gp120 (strains 51802, BORI, BJOX002, 254008, CNE20, TT31P.2792, B.6240, and A244), uncleaved trimeric gp140 (strains RHPA4259, AE.01.con_env03, 1086.C, C.CH505TF, WITO4160, BF1266, 9004S, and SC42261), and gp70-V1V2 scaffold proteins (strains B.CaseA, 7060101641, CM244.ec1, TV1.21, 001428.2.42, CAP210.2.00.E8, C2101.c01, BJOX002000.03.2, BF1266_431a, 96ZM651.02, RHPA4259.7, Ce1086_B2, 62357.14, 700010058, 191084_B7, and TT31P.2F10.2792), representing the global panel of HIV-1 Envs. Binding of sera to beads was detected using a secondary biotin-conjugated anti-rabbit IgG and measured via Bio-Plex. Binding is reported as AUC analysis values generated from measured mean fluorescent intensities (MFIs) at different serial dilutions. Criteria for positive reactivity was an MFI at 1:80 of >100, MFI at 1:80 of more than the antigen-specific cutoff (95th percentile of all prebleeds for study of each antigen), and MFI of more than 3-fold that of matched prebleeds before and after blank bead subtraction.

### ADCC assays.

ADCC activity was measured using a Luc-based assay against HIV-1 subtype B JRFL IMC-infected target cells performed as previously described (45). Target and effector cells were cocultured with serial dilutions of rabbit sera taken from weeks 0, 10, 18, and 34. Killing activity was detected by reduction in luciferase activity in each well. Results were considered positive if the percent specific killing was >15% after subtraction of activity observed at baseline. ADCC Ab titers were defined as the reciprocal of the highest dilution indicating a positive response.

### Statistical analysis.

Statistical analysis was performed using GraphPad Prism, v7.0. A two-tailed nonparametric *t* test (Mann-Whitney test) was used for all comparisons unless otherwise noted (*, *P* < 0.05). Spearman correlations were used to analyze relationships between multiple variables.
